# Vegetation index and livestock practices as predictors of malaria transmission in Nigeria

**DOI:** 10.1038/s41598-024-60385-z

**Published:** 2024-04-26

**Authors:** Oluyemi Okunlola, Segun Oloja, Ayooluwade Ebiwonjumi, Oyetunde Oyeyemi

**Affiliations:** 1 Department of Mathematics and Statistics, Redeemer’s University, Ede, Osun State Nigeria; 2 Department of Mathematical and Computer Sciences, University of Medical Sciences, Ondo City, Ondo State Nigeria; 3 Department of Mathematical Sciences, Adekunle Ajasin University, Akungba-Akoko, Ondo State Nigeria; 4 Department of Biosciences and Biotechnology, University of Medical Sciences, Ondo City, Ondo State Nigeria

**Keywords:** Malaria risk, Regression models, Vegetation, Livestock, Nigeria, Infectious diseases, Epidemiology

## Abstract

Nigeria is the most malaria-endemic country in the world. Vegetation and livestock practices have been linked to malaria transmission but little is known about these in Nigeria. The study aimed to evaluate the influence of vegetation and livestock as predictors of malaria transmission in Nigeria. Secondary data obtained from the Nigerian Demographic and Health Survey’s Geospatial Covariate Datasets Manual were used for the analysis. The survey was carried out successfully in 1389 clusters of thirty (30) households each using a two-stage stratified random sampling design. Hierarchical beta regression models were used to model the associations between malaria incidence, enhanced vegetation index (EVI), and livestock practices. The correlation coefficients for vegetation index and livestock-related variables ranged from − 0.063 to 0.074 and varied significantly with the incidence of malaria in Nigeria (*P* < 0.001). The model showed vegetation index, livestock goats, and sheep as positive predictors of malaria transmission. Conversely, livestock chicken and pigs were observed to reduce the risk of malaria. The study recommends the need to take into account local differences in transmission when developing malaria early warning systems that utilize environmental and livestock predictors.

## Introduction

Malaria is a disease with serious public health impacts and has significant implications on the quality of life and the economy^1^. The global cases of malaria and associated deaths were estimated at 247 million and 619,000 respectively, in 2021^2^. The African Region of the WHO bears a disproportionately large share of the global malaria burden. In 2021, the region had 95% of the malaria cases and 96% of the malaria deaths. The number of malaria deaths in Nigeria in 2021 is estimated at around 200,000, which is 31.9% of the worldwide total. Annually, over 60 million people are infected with malaria, and US$1.1 billion is lost due to malaria-related absenteeism and productivity losses^3^.

Over the years, malaria intervention programs implemented at both national and global levels have proven successful in reducing malaria transmission within the endemic regions of most sub-Saharan African countries^4^. While the impact of certain sociocultural and environmental variables in malaria transmission has been evaluated^5–7^, other possible factors such as vegetation index and livestock practices are inadequately addressed in many African countries including Nigeria.

Despite Nigeria being the most populated country in Africa and with rich vegetation diversity and index, few reports have identified vegetation index as a predictor of malaria risk^8^. As previous studies have shown, vegetation cover can affect malaria transmission and mosquito abundance by providing an outdoor resting place for the vector^9^. In addition, there is a dart of information on the impact of livestock on malaria transmission in the country. Nigeria is the highest producer of livestock in Africa and livestock contributed significantly to the country’s GDP in the third quarter of the year 2022^10^. The livestock sector is expected to undergo transformation as part of the Economic Recovery and Growth Plan, with the Agricultural Promotion Policy 2016–2020 prioritizing investments in the dairy and poultry sectors to commercialize output and close demand–supply gaps^11^. However, the long-term consequences of livestock sector change may have a severe influence on public health, environmental, and social outcomes due to some unknown and unpredictable elements defining its development trajectory^11^.

While transmission of zoonotic diseases is one of the most recorded public health implications of the livestock sector, the possibility of animal-human malarial transmission patterns is hardly emphasized in sub-Saharan African countries. As a result, malaria intervention programs are yet to integrate interventions in animals into the region’s operational implementation policies. Evidence from some malaria-endemic countries such as southern Tanzania, Indonesia, southern Malawi, and southwestern Ethiopia has revealed conflicting outcomes on the influence of livestock on malaria transmission and severity^12–15^. Specifically, certain medium-sized livestock such as goats, sheep, dogs, and pigs, and small-sized livestock like poultry have been reported to reduce malaria transmission^13,16^. There is evidence suggesting that cattle play a role in increasing mosquito density and the transmission of malaria^17^.

Unfortunately, no study is currently available in this regard in Nigeria despite being the most malaria-endemic country in the world and the highest livestock producer in Africa. Therefore, it becomes difficult to evaluate the contribution of livestock to malaria transmission in the country.

This study evaluated the contributions of vegetation index and livestock to the risk of malaria transmission in Nigeria. This becomes important as the study could provide useful information to support the current national policies on malaria control in Nigeria.

## Materials and methods

### Study area

The study was conducted in Nigeria. Nigeria is located between latitudes 4° 16′ and 13° 53′ North and longitudes 2° 40′ and 14° 41′ East. The country has a total land area of approximately 923,768 square kilometers and a population density of 212.04 people per square kilometer. Malaria is one of the country's most serious public health issues, and the country's climatic conditions make it ideal for recurrent malaria transmission^6^. In Nigeria, the common livestock animals are poultry birds, cattle, small ruminants (goats, sheep), pigs, and rabbits, and in some parts of the northern region of the country, donkeys, camels, and horses. The most commonly reared ones are chickens, cattle, goats, and sheep^18^. In the third quarter of 2022, the contribution of livestock production to Nigeria's GDP experienced an increase of 1.55 percent compared to the same period of the previous year^10^. Despite the close interaction between humans and livestock, their possible contribution to malaria transmission is yet to be explored in Nigeria.

### Data source and sampling procedures

The study relied on the Nigerian Demographic and Health Survey’s Geospatial Covariate Datasets Manual, in which certain variables in the survey were aggregated at the cluster level to facilitate the computation of indices, incidence, and prevalence. Data access was granted upon request for download via the DHS program website, https://dhsprogram.com/. Malaria incidence (MIN), enhanced vegetation index (EVI), and livestock ownership indices for cattle (LSCI), chicken (LSCH), goat (LSGO), sheep (LSSH) and pig (LSPI) were the variables of interest in this study. The dependent variable is MIN, while the other variables are predictors. The survey was carried out successfully in 1389 clusters of thirty (30) households each using a two-stage stratified random sampling design.

Malaria incidence was computed by calculating the average number of people per cluster who had clinical symptoms of *Plasmodium falciparum* malaria during the survey year. Surface maps of livestock distribution that provide global livestock densities that have been adjusted to match Food and Agriculture Organization (FAO) national estimates for the reference year 2006 were obtained from the 2007 Gridded Livestock of the World (GLW) database to obtain livestock indices^19^. The surface maps were created using improved and detailed sub-national livestock data (derived from various national census reports and livestock surveys), new and higher resolution predictor variables, and revised modeling methods that included a more systematic evaluation of model accuracy and the representation of uncertainties associated with predictions. Wint and Robinson^20^ go into more detail about the modeling method, whereas Benjamin et al.^21^ provided detailed information on the variables in the DHS geospatial covariates datasets manual. These materials can be consulted for additional information.

### Models

In this study, the evaluation of different livestock as risk factors for malaria incidence was carried out using a hierarchical Bayesian regression model which adjusted for the contextual and geographical effects in the data. The regression function was formulated as follows:

1 $$MIN =f\left(EVI, LSCA, LSCH, {\text{LSGO}},\mathrm{ LSPI},\mathrm{ LSSH}\right)$$

2 $$MIN = {\propto }_{0}+{\propto }_{1}EVI+{\propto }_{2}LSCA+{\propto }_{3}LSCH+{\propto }_{4}LSGO+{\propto }_{5}LSPI +{\propto }_{6}LSSH+ {e}_{i}$$

where ${\propto }_{i}$ are the coefficients of the linear model and ${e}_{i}$ stochastic error associated with the model.

Therefore, the general form of the linear model was obtained through the transformation of the above equations and expressed in the general form given as:

3 $$Y=X\beta +\in$$

However, the general linear model specification is not appropriate in this case because the response variable is bounded and takes values between 0 and 1. The transformation (3) in a spatial generalized linear mixed model is given as;

4 $$g\left(\mu \right)=X\beta +\varphi$$

The $g\left(\mu \right)$ is the link function, $X\beta$ is the linear predictor where $\varphi$ is the random effect term. The invertible link function, $g\left(\mu \right)$ is controlled by the statistical distribution of the response variable. As a custom in generalized linear model (GLM), the response variable must have its probability distribution in the exponential family. The dependent variable in the current study is a proportion and so a beta distribution was assigned and consequently logit link function was used. The probability density function and the logit link function are respectively given as;

5 $$f\left( y \right) = \frac{1}{{B\left( {\alpha ,\beta } \right)}}y^{\alpha - 1} \left( {1 - y} \right)^{\beta - 1} ,\,\,0 < y < 1,\,\,\alpha ,\beta > 0,\,\,B\left( {\alpha ,\beta } \right) = \frac{{\Gamma_{\alpha } \Gamma_{\beta } }}{{\Gamma_{\alpha + \beta } }}$$

6 $$log\frac{\lambda }{1-\lambda }=\eta =X\beta +\varphi$$

The symbol $\varphi$ was introduced to capture spatial variation because the data used in the study are measure at different spatial units (cluster in this case). This is in line with the common belief that measurements in neigbouring cluster have a tendency of having similar values of malaria incidence. The most popular way to represent spatial proximity in lattice data is to construct adjacent matrix in which two areas are defined to be spatially related if they have border that touch on the map and non-neighbour if there is no such relationship, resulting to a $n\times n$ binary matrix. Several approaches are available in disease mapping to accommodate geographical relationship disease mapping. This study use Leroux global prior, one of the most popular and efficient global priors. With this prior, the spatial dependence effects is the conditional expectation ${\varphi }_{c}$, that is,$E({\varphi }_{c}/{\varphi }_{-c})$. This is interpreted as the weighted average of the random effects in its adjacent clusters. The full conditional for all set $C$ random effects produce a unique Gaussian Markov Random Field (GMRF) with a multivariate normal distribution given as;

7 $$C\sim N(0, \sum )$$

The precision matrix, $\sum$ for the Leroux prior is defined as;

8 $${\sum }^{-1}=\frac{1}{\tau }\left({I}_{n}-\frac{\rho }{{\lambda }_{max}}G\right); \rho \epsilon [\mathrm{0,1})$$

The quantity $G$ and ${\lambda }_{max}$ in Eq. (8) are matrix and eigenvalue, respectively. To ensure correct model specification, the matrix $G$ is defined as follows;

9 $$G={I}_{n}-R;R=diag\left({n}_{i}\right)-W and {\lambda }_{max}=1$$

Substituting Eq. (8) into Eq. (9) and simplifying the resulting expression yields;

10 $${\sum }^{-1}=\frac{1}{\tau }\left({I}_{n}-\frac{\rho }{1}\right){I}_{n}-R=\frac{1}{\tau }\left({I}_{n}-\rho \left({I}_{n}-R\right)\right)=\frac{1}{\tau }\left(\left(1-\rho \right){I}_{n}+\rho R\right)$$

The hyper-parameters ${\theta }_{1}={\text{log}}(\tau )\sim logGamma(\mathrm{1,0.001})$ and ${\theta }_{2}=logit(\rho )\sim N(\mathrm{0,0.1})$

According to Gómez-Rubio^22^, “the structure of the precision matrix is a convex combination of an identity matrix ${I}_{n}$ (to represent $i.i.d$ spatial effect) and the precision of an intrinsic CAR $R$ (to represent a spatial pattern)”.

The model is implemented in R software using fast and accurate integrated nested Laplace approximation (INLA) through R-INLA package^23^. However, it should be noted that direct implementation of the model was not possible in R-INLA, but was achieved through the “generic1” latent effect with the condition that the first expression in Eq. (9) holds.

## Results

**Figure 1:**
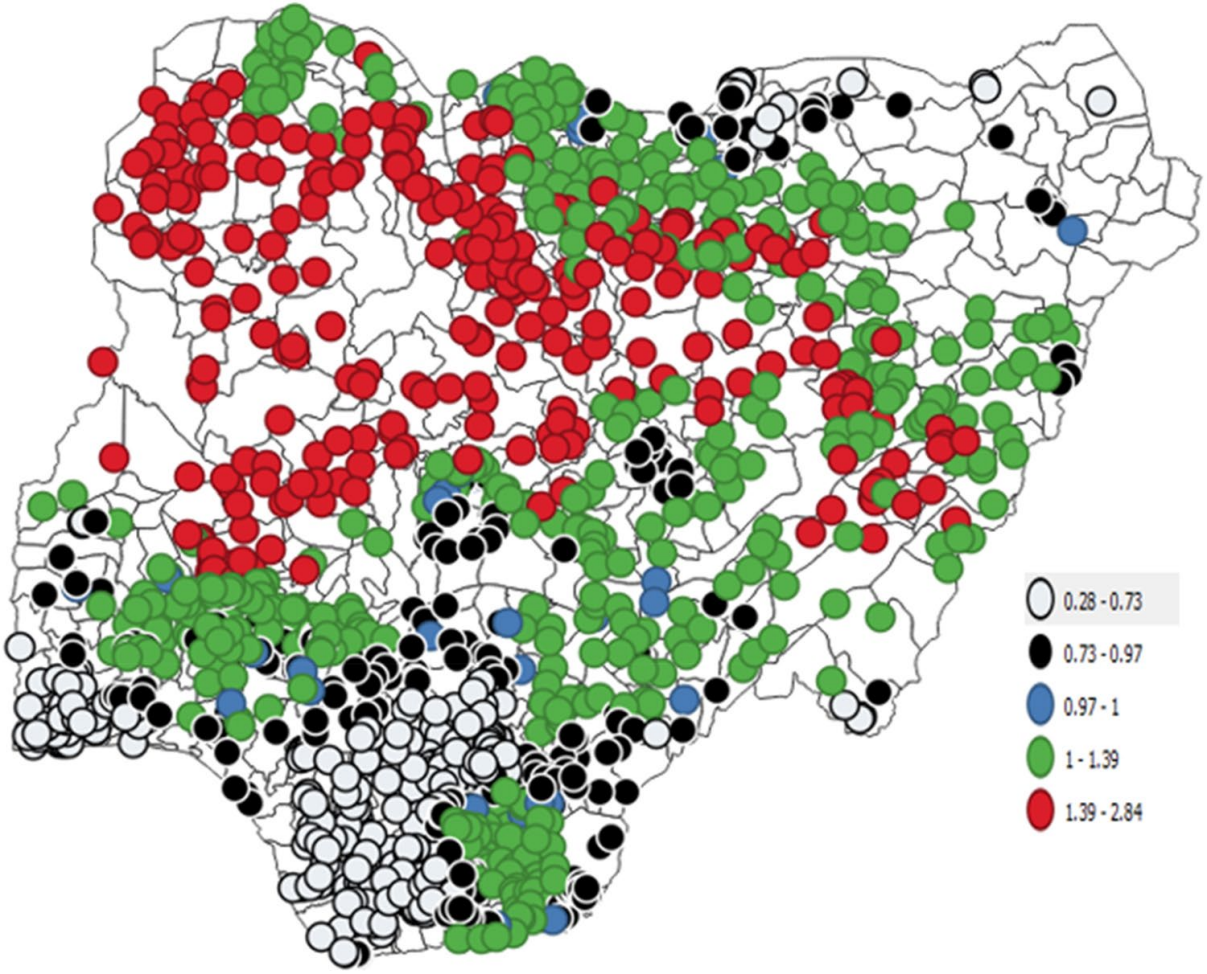
Map of relative risk of Malaria incidence based on the hierarchical beta regression.

Table 1 shows the descriptive analysis of the variables such as malaria incidence (MIN), enhanced vegetation index (EVI), livestock cattle (LSCA), chicken (LSCH), goat (LSGO), pig (LSPI), and sheep (LSSH) under investigation in this study. The mean values of MIN, EVI, LSCA, LSCH, LSGO, LSPI, and LSSH are presented in Table 1. The correlation coefficients for enhanced vegetation index and livestock-related variables ranged from − 0.063 to 0.074 and varied significantly with the incidence of malaria in Nigeria (*P* < 0.001) (Table 2). The low-level values of the correlation coefficient between pairs of independent variables indicated the absence of multicollinearity, a serious problem in regression analysis. The posterior means and the 95% credible interval of each covariate and the intercept term is presented in Table 3. The exponentiated quantity can be interpreted as the relative risk of malaria incidence attributed to the specific independent variable. Consequently, an increase of 1 unit in livestock cattle, chicken, and pig is associated with a decrease of 0.03%, 3%, and 0.26% risk of malaria incidence, respectively. Whereas, an increase of 1-unit enhanced vegetation index, livestock goats and sheep are associated with corresponding increases of 85%, 0.1%, and 2% risk of malaria incidence, respectively. The specific relative risk of malaria incidence of each location considered in the study is mapped in Fig. 1. The gray, black, blue, green, and red bubbles are used to depict locations with a very low, low, moderate, high, and very high residual relative risk.

**Table 1:** Descriptive analysis.

	MIN	EVI	LSCI	LSCH	LSGO	LSSH	LSPI
Mean	0.351	3131.506	14.923	376.245	64.195	39.040	13.622
Median	0.362	3209.333	4.521	205.473	34.671	8.604	1.018
Maximum	0.611	4962.000	323.468	6224.752	1324.266	525.382	454.892
Minimum	0.097	1131.583	0.106	0.217	0.257	0.051	0.000
Std. dev	0.091	795.800	25.115	624.046	99.307	68.185	37.231
Skewness	− 0.277	0.068	4.524	4.712	6.120	2.797	6.305
Kurtosis	− 0.195	− 1.030	37.994	28.459	55.063	8.900	53.048

*MIN* Malaria incidence, *EVI* enhanced vegetation index, *LSCI* livestock cattle, *LSCH* livestock chicken, *LSGO* livestock goat, *LSSH* livestock sheep, *LSPI* livestock pigs.

**Table 2:** Correlation between malaria incidence and predictors of transmission.

Variable	MIN	EVI	LSCI	LSCH	LSGO	LSSP	LSPI
MIN	1						
EVI	− 0.063*	1					
LSCI	0.093**	− 0.470**	1				
LSCH	− 0.205**	0.249**	− 0.079**	1			
LSGO	− 0.004	− 0.310**	0.532**	0.129**	1		
LSSP	0.074**	− 0.503**	0.685**	− 0.069*	0.540**	1	
LSPI	0.000	0.124**	− 0.060*	0.054	− 0.090**	− 0.158**	1

*MIN* Malaria incidence, *EVI* enhanced vegetation index, *LSCI* livestock cattle, *LSCH* livestock chicken, *LSGO* livestock goat, *LSPI* livestock pigs, *LSSH* livestock sheep.

**Table 3:** Result of spatial generalized linear mixed beta regression.

	Mean	SD	0.5quant	0.025quant	0.975quant	kld
Intercept	− 5.47504	0.61421	− 6.73936	− 5.47360	− 4.22147	0
EVI	0.61647	0.04375	0.53066	0.61647	0.70227	0
LSCI	− 0.00035	0.00925	− 0.01849	− 0.00035	0.01779	0
LSCH	− 0.02910	0.00815	− 0.04509	− 0.02910	− 0.01310	0
LSGO	0.00123	0.00944	− 0.01729	0.00123	0.01975	0
LSSP	0.01958	0.00923	0.00148	0.01958	0.03767	0
LSPI	− 0.00265	0.00333	− 0.00919	− 0.00265	0.00389	0

*MIN* Malaria incidence, *EVI* enhanced vegetation index, *LSCI* livestock cattle, *LSCH* livestock chicken, *LSGO* livestock goat, *LSSH* livestock sheep, *LSPI* livestock pigs,

## Discussion

Despite considerable resources invested in malaria control programs in Nigeria, their success has been limited^24^. Between 2017 and 2020, malaria cases increased by 5.3%, with a corresponding 4.7% rise in associated deaths among the at-risk population^25^. While environmental variables influencing malaria transmission have been somewhat explored in Nigeria, the impact of vegetation index remains poorly understood. To our knowledge, no study has assessed the relationship between livestock and malaria transmission in Nigeria, despite reported associations elsewhere in sub-Saharan Africa^12,14,15^.

Our study reveals vegetation index as a positive predictor of malaria transmission. This is consistent with findings from Eritrea, Ethiopia, and Uganda^26–28^. In addition to commonly assessed factors like temperature and rainfall, vegetation cover has emerged as a significant factor influencing malaria transmission, potentially by providing resting places for mosquito vectors^29^. The dense vegetation structure characteristic of tropical rainforests in southern Nigeria may contribute to heightened malaria transmission in the region. Conversely, in regions with limited moisture content, such as some parts of northern Nigeria, vegetation greenness may serve as an indirect indicator of rainfall, facilitating mosquito breeding and increasing malaria transmission risk^27^. Consequently, the interplay of vegetation and other drivers identified in our previous studies likely contributes to the widespread incidence of malaria across Nigeria's regions^6^.

The strategy of using environmental measures to mitigate malaria transmission, rooted in the concept of zooprophylaxis—redirecting malaria vectors away from humans—has long been advocated^30^. However, this approach can inadvertently heighten human exposure to malaria if it increases opportunities for mosquitoes to feed on alternative hosts. By fostering greater numbers of animals near mosquito breeding sites, blood meals become more accessible, potentially attracting more mosquitoes, enhancing their survival, and elevating the risk of disease transmission to humans, a phenomenon termed zoopotentiation^12^. Regional variations in these concepts have led to conflicting observations.

Our study, employing spatial beta regression models as detailed in Table 3, identifies livestock goats and sheep as positive predictors of malaria transmission in Nigeria, while livestock cattle, chicken, and pigs emerge as negative predictors. This suggests that the concept of zoopotentiation may apply to goats and sheep as malaria risk factors, although the impact of sheep was not statistically significant in this analysis. As previously noted, the presence of these livestock may elevate the relative abundance of mosquitoes carrying *Plasmodium* species^31^. The varied host selection by vectors targeting these livestock could heighten the potential for human exposure to *Plasmodium*. Moreover, keeping animals within or near households can attract more vectors due to the odors and heat they emit^12^. Furthermore, physical disturbances caused by large livestock such as cattle, such as puddles and hoof prints, may exacerbate zoopotentiation by creating additional larval habitats and thereby increasing adult vector density in close proximity to human residences^13^.

The significant negative influence of livestock cattle on malaria transmission underscores their potential for zooprophylaxis. The study by Finney et al.^32^ highlights that cattle and pigs contribute a notably higher proportion of blood meals compared to other livestock. Therefore, the heightened blood meal availability in cattle, pigs, and chickens, identified as having zooprophylactic potential, can reduce the risk of human exposure to malaria, as previously observed in larger livestock like cattle^33^. The zooprophylactic role of livestock in malaria risk reduction has also been documented in Malawi and Zambia^11,34^.

Advocacy for using livestock as bait to attract mosquitoes has emerged as a promising alternative to insecticide adoption. However, integrating zooprophylaxis with insecticide-treated livestock becomes more pertinent in areas where zoophilic vectors transmit the malaria parasite^31^. This approach aids in vector control without exacerbating the issue of mosquito resistance to insecticides^35^.

In this study, we employed hierarchical beta regression to forecast the impact of livestock on malaria transmission. This choice was driven by the necessity to incorporate the spatial aspect of the data and account for the response variable (malaria incidence), which is a proportion ranging between 0 and 1. Ordinary least squares regression, typically used for continuous responses, would be inappropriate in this context due to the nature of the response variable, resulting in inefficient parameter estimates.

## Conclusion

This study examines the influence of vegetation and livestock on malaria transmission. The findings consistently indicate that the vegetation index increases the risk of malaria transmission in Nigeria in the tested model. Certain livestock species, such as goats and sheep, are identified as positive predictors of malaria transmission, whereas livestock like cattle, pigs, and chicken are negative predictors, suggesting their potential for prophylactic use.

The study underscores the importance of considering local variations in transmission patterns when devising malaria early warning systems that integrate environmental and livestock factors. Such tailored approaches can lead to more accurate early warning systems, crucial for effective malaria control. For instance, one potential strategy that can be recommended is insecticide-treated livestock into zooprophylaxis in areas where mosquitoes can feed on both animal and human hosts, thereby enhancing malaria control efforts. One limitation of the study lies in the utilization of secondary data for the findings, which may not fully extrapolate to real-life scenarios due to potential biases or constraints inherent in the dataset. Thus, it's suggested that a randomized controlled trial be conducted to authenticate and substantiate the modeling outcomes of livestock and malaria transmission. This approach would offer a more robust and reliable validation of the study's results, ensuring greater confidence in their applicability to practical settings.

## Data Availability

All data are included in the manuscript and further queries about sharing data can be directed to the corresponding author.
